# Micro-RNA-21 (biomarker) and global longitudinal strain (functional marker) in detection of myocardial fibrotic burden in severe aortic valve stenosis: a pilot study

**DOI:** 10.1186/s12967-016-1011-9

**Published:** 2016-08-27

**Authors:** Iacopo Fabiani, Cristian Scatena, Chiara Maria Mazzanti, Lorenzo Conte, Nicola Riccardo Pugliese, Sara Franceschi, Francesca Lessi, Michele Menicagli, Andrea De Martino, Stefano Pratali, Uberto Bortolotti, Antonio Giuseppe Naccarato, Salvatore La Carrubba, Vitantonio Di Bello

**Affiliations:** 1 Department of Surgical, Medical, Molecular Pathology and Critical Area, Cisanello Hospital, University of Pisa/A.O.U.P, Via Paradisa, 2, 56100 Pisa, PI Italy; 2 Department of Translational Research and New Technologies in Medicine and Surgery, University of Pisa, 56100 Pisa, Italy; 3 Fondazione Pisana per la Scienza, 56100 Pisa, Italy; 4 Division of Cardiac Surgery, Department of Surgical, Medical, Molecular Pathology and Critical Area, Cisanello Hospital, University of Pisa/A.O.U.P, 56100 Pisa, Italy; 5 Division of Internal Medicine, Villa Sofia Hospital, 90100 Palermo, Italy

**Keywords:** Aortic valve, Aortic stenosis, Aortic valve replacement, Tissue characterization, Tissue and strain Doppler echocardiography, Myocardial strain

## Abstract

**Aims:**

Myocardial fibrosis (MF) is a deleterious consequence of aortic valve stenosis (AVS). Global longitudinal strain (GLS) is a novel left ventricular (LV) functional parameter potentially useful to non-invasively estimate MF. MicroRNAs (miRNAs) are non-coding small ribonucleic acids (RNA) modulating genes function, mainly through RNA degradation. miRNA-21 is a biomarker associated with MF in pressure overload. The aim of the present study was to find an integrated algorithm for detection of MF using a combined approach with both bio- and functional markers.

**Methods:**

Thirty-six patients (75.2 ± 8 y.o.; 63 % Female) with severe AVS and preserved LV ejection fraction (EF), candidate to surgical aortic valve replacement (sAVR) were enrolled. Clinical, bio-humoral evaluation (including plasmatic miRNA-21 collected using specific tubes, PAXgene, for stabilization of peripheral RNA) and a complete echocardiographic study, including GLS and septal strain, were performed before sAVR. Twenty-eight of those patients underwent sAVR and, in 23 of them, an inter-ventricular septum biopsy was performed. Tissues were fixed in formalin and embedded in paraffin. Sections were stained with Hematoxylin and Eosin for histological evaluation and with histochemical Masson trichrome for collagen fibers. The different components were calculated and expressed as micrometers^2^. To evaluate tissue miRNA components, sections 2-μm thick were cut using a microtome blade for each slide. Regression analysis was performed to test association between dependent variable and various predictors included in the model.

**Results:**

Despite a preserved EF (66 ± 11 %), patients presented altered myocardial deformation parameters (GLS −14,02 ± 3.8 %; septal longitudinal strain, SSL −9.63 ± 2.9 %; septal longitudinal strain rate, SL-Sr −0.58 ± 0.17 1/s; Septal Longitudinal early-diastolic strain rate, SL-SrE 0.62 ± 0.32 1/s). The extent of MF showed an inverse association with both GLS and septal longitudinal deformation indices (GLS: R^2^ = 0.30; p = 0.02; SSL: R^2^ = 0.36; p = 0.01; SL-Sr: R^2^ = 0.39; p < 0.001; SL-SrE: R^2^ = 0.35; p = 0.001). miRNA-21 was mainly expressed in fibrous tissue (p < 0.0001). A significant association between MF and plasmatic miRNA-21, alone and weighted for measures of structural (LVMi R^2^ = 0.50; p = 0.0005) and functional (SSL R^2^ = 0.35; p = 0.006) remodeling, was found.

**Conclusions:**

In AVS, MF is associated with alterations of regional and global strain. Plasmatic miRNA-21 is directly related to MF and associated with LV structural and functional impairment.

## Background

Aortic valve stenosis (AVS) is the most common valvular heart disease in Western Countries [1, 2]. In particular, AVS is a slowly progressive disease, associated with significant Left Ventricular (LV) pressure overload, which induces left ventricular hypertrophy (LVH) and secondary myocardial fibrosis (MF). MF is an early morphologic alteration in patients with AVS and a major determinant of LV functional impairment, ultimately leading to the development of heart failure.

Accordingly, robust and repeatable measures of MF that may be applied in the clinical field are eagerly awaited.

Nevertheless, the evaluation of patients with AVS is generally limited to the assessment of flow-dependent parameters (velocity; gradients) that reflect only the “valvular side” of the pathology, disregarding LV components of disease [3–5].

Beside the endo-myocardial biopsy (gold standard), not ethically feasible in a clinical setting, a series of biomarkers and cardiac imaging techniques have been lately proposed to combine tissue parameters with functional evaluation. In this respect, the evaluation of LV deformation by Speckle Tracking Echocardiography (2D-STE) has been shown to allow a better assessment of cardiac contractile function than traditional parameters [i.e. Ejection Fraction (EF)], giving the chance to assess the presence of subtle alterations of LV systolic performance. In particular, regional and global longitudinal strain (GLS), showed a better and earlier diagnostic power over EF, becoming reference indicators for the precocious assessment of sub-clinical LV functional impairment [6–12]. More recently, measures obtained through 2D-STE showed to correlate with the presence and extent of MF at cardiac magnetic resonance, as assessed through T1-mapping and late gadolinium enhancement as quantification techniques [13, 14].

MicroRNAs (miRNAs) are non-coding small ribonucleic acids (RNAs) that modulate the expression of target genes inducing mRNA degradation. The expression of miRNAs is associated with multiple pathological processes that affect also the cardiovascular system [15, 16].

A regulatory role for miRNA-21 has been evidenced in LV myocardial remodeling induced by hemodynamic stress [17, 18].

Recent reports indicate that the presence of circulating miRNAs may reflect specific cardiovascular pathologies and could be a useful biomarker for different cardiovascular diseases.

Accordingly, the evaluation of both miRNA (as a biomarker) and GLS (as a functional marker), might allow an integrated assessment of the pathophysiological relationship between MF and adverse LV remodeling.

With these considerations in mind, we aimed at assessing:

- the presence of MF (detected by endo-myocardial biopsy) both with gold standard histologic method and with an advanced laser micro-dissection methodology (tissue miRNA-21), in patients with severe AVS;
- the presence of a direct association between plasmatic and tissue pool of miRNA-21;
- the relationship between 2D-STE parameters, MF (endo-myocardial biopsy) and plasmatic/tissue miRNA-21 expression levels, in order to develop a non-invasive detection of myocardial fibrotic burden.

## Methods

### Study population

Thirty-six consecutive patients with severe symptomatic AVS (Peak Trans-valvular Velocity > 4 m/s; Mean Gradient > 40 mmHg; Aortic Valve Area-AVA < 1 cm^2^; AVAi < 0.6 cm^2^/m^2^) and preserved EF, were prospectively evaluated in University of Pisa Hospital (A.O.U.P) for sAVR. Patients underwent laboratory analysis (for 36 patients brain natriuretic peptide, BNP pg/mL; high-sensitive assays troponin T, hs-TnT, ng/L; plasmatic miRNA-21 assay for 30 patients), and trans-thoracic echocardiography (M-Mode, 2D, Doppler, Tissue Doppler Imaging-TDI, 2D-STE). Twenty-eight patients were finally submitted to sAVR (three patients refused surgery; five patients decision for percutaneous procedures), 23 of whom (in 5 patients under-sampling/biopsy not performed) underwent intra-operatory basal inter-ventricular septum biopsy to evaluate MF (23 patients) and tissutal levels of expression of miRNA-21 (20 patients). All patients signed an informed consent, approved by local ethical committee, conform to the ethical guidelines of the 1975 Declaration of Helsinki. We excluded patients with at least one of the following: age < 18 y.o., significant major comorbidities (i.e.cancer; dialysis; cachexia), inability to sign consent, pregnancy, poor acoustic window, ischemic heart disease (including epicardial coronary artery disease > 50 %), associated valvular disease of moderate-severe degree, non-degenerative AVS, diskynetic septum (i.e. stimulator; Left Bundle Branch Block).

### Conventional echocardiography

Transthoracic exams were performed with a dedicated machine (Vivid-7, General Electric Milwaukee, WI-USA). Patients were imaged in the left lateral decubitus position and data were acquired with a 4 MHz (M4S) transducer at a depth of 16 cm in the parasternal (long- and short-axis views) and apical views (two- and four-chamber and apical long-axis views). All parameters were derived according to current indications, and considered in relation to their established reference ranges [19, 20].

LV dimensions were calculated from the standard M-mode/2D images at the parasternal long-axis views and included LV diameters and end-diastolic thickness of the interventricular septum and posterior wall. Left ventricular mass was calculated and corrected by the body surface area to derive mass index (LVM_i_). The LV end-diastolic and end-systolic volumes were measured from the apical two- and four-chamber views, and EF was calculated using the Simpson’s rule. LV diastolic function was evaluated using early (E wave) and late (A wave) trans-mitral velocities, the E/A ratio, and the E wave deceleration time obtained from the spectral pulsed-wave Doppler recordings. In addition, TDI was performed, adjusting gain and frame rate to get an appropriate tissue characterization. The aortic valve area (AVA; indexed, AVAi) was calculated by the continuity equation, and the maximum pressure gradient across the restrictive orifice was estimated by the modified Bernoulli equation. Mean trans-aortic pressure gradient was calculated averaging the instantaneous gradients over the ejection period on the continuous-wave Doppler recordings. As a measurement of global left ventricular afterload, the valvulo-arterial impedance (Z_VA_) was calculated. Finally, color Doppler echocardiography was performed after optimizing gain and Nyquist limit in order to evaluate the presence of regurgitant valve disease. The severity of valvular regurgitation was determined on a qualitative scale (mild, moderate, and severe), according to the current guidelines [19, 20].

### Speckle tracking echocardiography

Assessment of LV GLS was performed using 2D-STE (frame rate 45–90 frame/s, fps). We limited the analysis to the global longitudinal component of strain (peak value-mid myocardium). Quantifications were performed using the available software (EchoPAC 10, General Electric), as described previously [21–23]. For this purpose, standard 2D grey-scale images of the LV were acquired at conventional apical two- and four-chamber and apical long-axis views. 2D-STE enables angle-independent myocardial deformation analysis by tracking frame-to-frame natural acoustic markers, or speckles, that appear equally distributed within the myocardial wall. Applying the strain Lagrangian formula, the percentage change in myocardial length relative to the initial length derives myocardial strain (expressed in percentage). The temporal derivation of myocardial strain results in strain rate and is a measure of the rate of deformation. The longitudinal deformation relates to motion from mitral annulus to the apex in the apical views and results in shortening (negative strain) and lengthening (positive strain). Using the dedicated application, the endocardial contour was manually traced at an end-systolic frame. The software then automatically traced a concentric region of interest (ROI), including the entire myocardial wall. The myocardial tracking was verified, and the ROI width was adjusted to optimize the tracking, if needed. Next, segmental strain analysis was performed dividing each LV image into six segments. Peak systolic parameters were calculated averaging the peak systolic values of the eighteen segments, derived from the six segments of the three apical views (two- and four-chamber and apical long-axis views). For dedicated septal analysis, a focused ROI (80–95/fps) was traced specifically for inter-ventricular septum. We assessed, septal longitudinal systolic strain (SSL), systolic (SL-Sr) and early-diastolic (SL-SrE) strain rates. We then averaged measure from anterior and inferior septum. Intra and inter-observer variability analysis for 2D-STE was evaluated by intra/inter-class correlation coefficient (ICC). Ten randomly selected patients were evaluated three-times by the same operator (same beats; consecutive days). The same measurements were repeated in the same day by a second clinician, blinded to previous results. All ICC resulted >0.80 (p < 0.05), showing good agreement.

### Invasive measurements

In 22 patients, in addition to coronary angiography, standard left heart catheterization was performed before sAVR. Peak-to-peak gradient, invasive end-diastolic pressure (EDPi; fluid-filled catheter) and semi-quantitative aortic regurgitation were evaluated.

### Operating myocardial biopsy

In 23 patients undergoing sAVR, concomitant intra-operatory basal left-side inter-ventricular septum biopsy was performed to assess MF, as previously described [24–26]. Briefly, tissues (30–80 mg) were fixed in 10 % formalin and embedded in paraffin. One Section (2 µm) was stained with Hematoxylin and Eosin for histological evaluation and one Section (5 µm) with histochemical Masson trichrome stain for collagen fibers. The different components of the myocardial biopsy were calculated by computer analysis (PALM MicroBeam, Carl Zeiss), and expressed as micrometers squared. In particular, the following parameters were analyzed:

- the overall myocardial area occupied by the myocytes and connective tissue (fibrotic area);
- myocyte area
- connective area
- connective/overall myocardial area (ratio % as MF grading)

All measurements were made by two expert pathologists without knowledge of the clinical data (intra-class and inter-observer correlation coefficients on 5 random samples of 0.9 and 0.94, respectively).

### Origin of myocardial miRNA-21

#### Immunohistochemistry

4-micron sections were dewaxed in xylene and rehydrated through graded alcohols to water. Antigen retrieval was performed microwaving sections for 9 min in citrate/EDTA buffer (pH 7.8). Not specific peroxidase activity was blocked with 3 % hydrogen peroxidase for 15 min, and non-specific binding prevented by incubation with normal goat serum for 10 min. Afterwards, incubation with anti-vimentin mouse monoclonal antibody (clone VI10, Abcam, diluition 1:200) and anti-CD45/LCA rabbit monoclonal antibody (clone EP68, Cell Marque, diluition 1:100) was performed for 1 h at room temperature. A biotin conjugated goat derived secondary antibody was applied followed by the Vectastain Elite ABC kit (Vector Laboratories). Slide were incubated with diaminobenzidine tetrahydrochloride (DAB) and counterstained with hematoxylin.

### miRNA-21: plasmatic and tissue study

#### Blood samples

 Peripheral blood samples were collected using specific tubes (PAXgene Blood RNA Tube) included in the commercial systems for collection and immediate stabilization of peripheral blood RNA (PreAnalytiX).

#### Tissue samples

For 20 biopsy specimens laser microdissection (LSMD) was performed. Sections 2-μm thick were cut from each case using a new microtome blade for each slide. The PALM MicroBeam laser micro-dissector from Carl Zeiss was used to select and collect cardiomyocytes and fibrotic cells to be studied separately.

#### RNA isolation

Blood RNA was purified using the commercial kit PAXgene Blood RNA Kit (PreAnalytiX). The quantity of extracted RNA was estimated with Qubit 2.0 Fluorometer (Life Technologies) by using 2ul of undiluted RNA solution. Yield ranged from 50 to 500 ng/ul of RNA. Microdissected samples were incubated at 55 °C overnight upside-down with 50 μl of lysis buffer and 10 μl of proteinase K. The day after the samples were loaded in Maxwell 16 Instrument (Promega) to extract RNA.

#### Reverse transcription and analysis of miRNA profiles

MicroRNAs were reverse transcribed from 6 µl of total extracted RNA sample using the miScript II RT Kit (QIAGEN). cDNA from micro dissected samples was pre-amplified prior to real-time PCR analysis of miRNA. miRNA expression analysis were performed in triplicate using 1 µl of diluted cDNA as a template for real-time PCR with the miScript SYBR Green PCR Kit (QIAGEN) and the miScript Primer Assays (SNORD61—assay code MS00033705, miRNA-21—assay code MS00009079 (QIAGEN)) according to manufacturer’s instructions on the CFX96 Real Time system c1000 thermal cycler (BIORAD).

### Data analysis

Data analysis was performed using the Bio-Rad CFX Manager Software v3.1 and Microsoft Excel. miRNA 21 expression was calculated using SNORD61 expression level as reference and the relative normalized expression ∆∆Cq formula.

### Statistical analysis

The data sets were assessed for normality with the Kolmogorov–Smirnov test. Continuous variables are described as mean ± standard deviation (SD). Otherwise as median (with minimum/maximum). Categorical data are reported as percentage. Plasmatic and tissue miRNA-21 levels of expression where measurable were treated as continuous variables. Continuous variables were compared using the Mann–Whitney *U* test when non-Gaussian. Univariate linear regression analysis was performed to test association between dependent variable and various potential predictors included in the model. We assessed also the association between Myocardial Fibrosis (Y) and Plasmatic miRNA-21 (X) weighted for left ventricular mass indexed for BSA and septal longitudinal strain, respectively. The threshold for statistical significance was p < 0.05. In order to preserve the statistical meaning of regression analysis (direct/inverse correlation/association), in the text we considered global longitudinal strain/systolic strain rate in absolute value. Using a c-statistic approach we derived the miRNA-21 value with the best combination of sensitivity and specificity for discrimination of patients with a significant amount of MF (more than 10 % of the specimen).

The following statistic package was used: Medcalc 12.7 (Medcalc Software 2013, Belgium).

## Results

 The characteristics of the population regarding clinical, laboratory and echocardiographic parameters are shown in Tables 1, 2 and 3.

**Table 1:** Population characteristics

	Mean	SD
*General characteristics*		
Age (year)	75.2	8.06
BSA (m^2^)	1.8	0.17
Log EUROSCORE (%)	5.9	4.17
EUROSCORE II (%)	2.2	1.13
SAP (mmHg)	139.1	19.00
DAP (mmHg)	71.3	10.03
HR (bpm)	73.5	11.92

	n	%
*Clinical characteristics*		
Female sex	23	63
CHD (</=50 % epicardial coronary)	12	33
COPD	8	22
Anemia	14	38
Chronic kidney dis.	22	61
Diabetes Mell.	8	22
Arterial hypertension	31	86
Dyslipidemia	21	58

	n	%
*Drugs (admission)*		
ACE-inhibitors	15	41
AT-II-inhibitors	10	27
Anti-aldosteronic	2	5
Diuretics	14	38
Calcium-antagonist	6	10

Laboratory data	Mean/Median*	SD/Min–Max**
BNP (pg/mL)	250.9	220.4
GFR (mL/min/1.73 m^2^)	70.8	28.4
hsTnT (ng/L)	30.4	26.8
miRNA-21 (30 pts)	2.02*	0.02–11.26**

*ACE* angiotensin converting enzyme, *AT-II* angiotensin 2 receptor, *BNP* brain natriuretic peptide, *BSA* body surface area, *CHD* coronary heart disease, *COPD* chronic obstructive pulmonary disease, *DAP* diastolic arterial pressure, *GFR* glomerular filtration rate, *HR* heart rate, *SAP* systolic arterial pressure

**Table 2:** Echocardiographic and invasive data

	Mean	SD
*Valvular parameters*		
AVAi (cm^2^/m^2^)	0.45	0.09
Max gradient (mmHg)	80.2	16.76
Mean gradient (mmHg)	49.7	7.67
Peak-peak gradient (mmHg)	58.3	15.40
Velocity-ratio	0.18	0.04
Peak velocity (m/sec)	4.4	0.34
*Non invasive haemodynamic data*		
SVi (mL/m^2^)	38.5	16.08
CI (L/min/m^2^)	2.5	0.72
CO (L/min)	4.8	1.45
Z_VA_ (mmHg/ml/m^2^)	5.9	1.26
*Diastolic function parameters*		
EDPi (mmHg) (invasive)	16.81	6.81
LAVi (mL/m^2^)	48.2	12.65
E/A	0.8	0.33
E/e’ Average	18.4	8.39
DT (msec)	247.2	93.25
*Conventional systolic function parameters (with TDI)*		
EF %	65.8	10.94
FS %	36.4	7.89
MAPSE (mm)	9.5	1.84
s’ l (cm/s)	6.4	1.50
s’s (cm/s)	5.6	1.49
*Left and right ventricular echo parameters*		
EDDi (cm/m^2^)	2.52	0.25
EDVi (mL/m^2^)	50.28	12.95
ESDi (cm)	1.71	0.30
ESVi (mL/m^2^)	17.47	8.55
LVMi (g/m^2^)	149.5	20.7
RWT	0.51	0.07
sPAP (mmHg)	30.8	6.46
TAPSE (cm)	1.8	0.25

*AVAi* indexed aortic valve area, *CI* cardiac index, *CO* cardiac output, *DT* deceleration time, *E/A* ratio of early to late diastolic mitral filling velocity, *E/e′* ratio of early diastolic velocity (PW) to tissue proto-diastolic velocity (TDI), *EDDi* indexed left ventricular end-diastolic diameter, *EDPi* invasive left ventricular end-diastolic pressure, *EDVi*, indexed left ventricular end-diastolic volume, *EF*, ejection fraction, *ESDi*, indexed left ventricular end-systolic diameter, *ESVi*, indexed left ventricular end-systolic volume, *FS*, fractional shortening, *LAVi*, indexed left atrial volume, *LVMi*, indexed left ventricular mass, *MAPSE*, mitral annular plane systolic excursion, *RWT*, relative wall thickness, *s’L*, systolic velocity (TDI) lateral, *s’S*, systolic velocity (TDI) septal, *sPAP*, systolic pulmonary arterial pressure, *SVi*, indexed stroke volume, *TAPSE*, tricuspid annular plane systolic excursion, *Z*_*VA*_, valvulo-arterial impedance

**Table 3:** Speckle tracking and tissue data

	Mean	SD
*Speckle tracking*		
GLS %	−14.02	3.88

	Mean/median*	SD/min–max**
*Septal speckle tracking and tissue data (septum)*		
SL-Sr (1/sec)	−0.58	0.17
SL-SrE (1/sec)	0.62	0.32
SSL (%)	−9.63	2.97
MF % (n.23)	18.45*	5.13–98.0**
miRNA-21 myocardial expression/myocardial area (n.20)	0.416*	0.05–1.53**
miRNA-21 fibrotic/fibrotic area (n.20)	4.041*	0.57–22.27**

*GLS* global longitudinal strain, *MF* myocardial fibrosis, *miRNA* micro-RNA, *SL-Sr* septal systolic strain rate, *SL-SrE* septal early-diastolic strain rate, *SSL* septal longitudinal strain

All patients (Tables 2 and 3) showed a significantly elevated left ventricular mass indexed (LVMi) for body surface area (BSA), with evidence of concentric LV hypertrophy. A variable degree of diastolic impairment was observed, with increased EDPi and left atrial dimensions. LVMi didn’t show a significant association with indices of AVS severity (AVAi; Max/mean gradients; peak-to-peak gradient; Velocity Ratio; Z_VA_). Even if conventional indices of global systolic function were preserved (EF, Fractional shortening), more sensitive parameters of longitudinal function (e.g. Mitral annular plane systolic excursion, MAPSE; TDI; Table 2) were reduced when compared to normal ranges.

### Speckle tracking analysis

A significant impairment of global longitudinal deformation parameters (GLS) was observed (Table 3).

In particular, GLS showed a direct relationship with indexed LV stroke volume (SVi) (R^2^ = 0.20; p = 0.006) and a significant inverse relationship with BNP levels (R^2^ = 0.47; p = 0.007). Modest inverse relationships between LVMi and AVAi (R^2^ = 0.16; p = 0.01), Z_VA_ (R^2^ = 0.12; p = 0.03) and GLS (R^2^ = 0.23; p = 0.002), were also observed. Septal sub-analysis showed higher impairment of deformation indices and a significant direct relationship of SSL with stroke volume (R^2^ = 0.22; p = 0.003).

### Tissue analysis: myocardial fibrosis

A variable amount of MF (with absence of inflammatory cells) was a common finding in patients who performed biopsy.

**Fig. 1:**
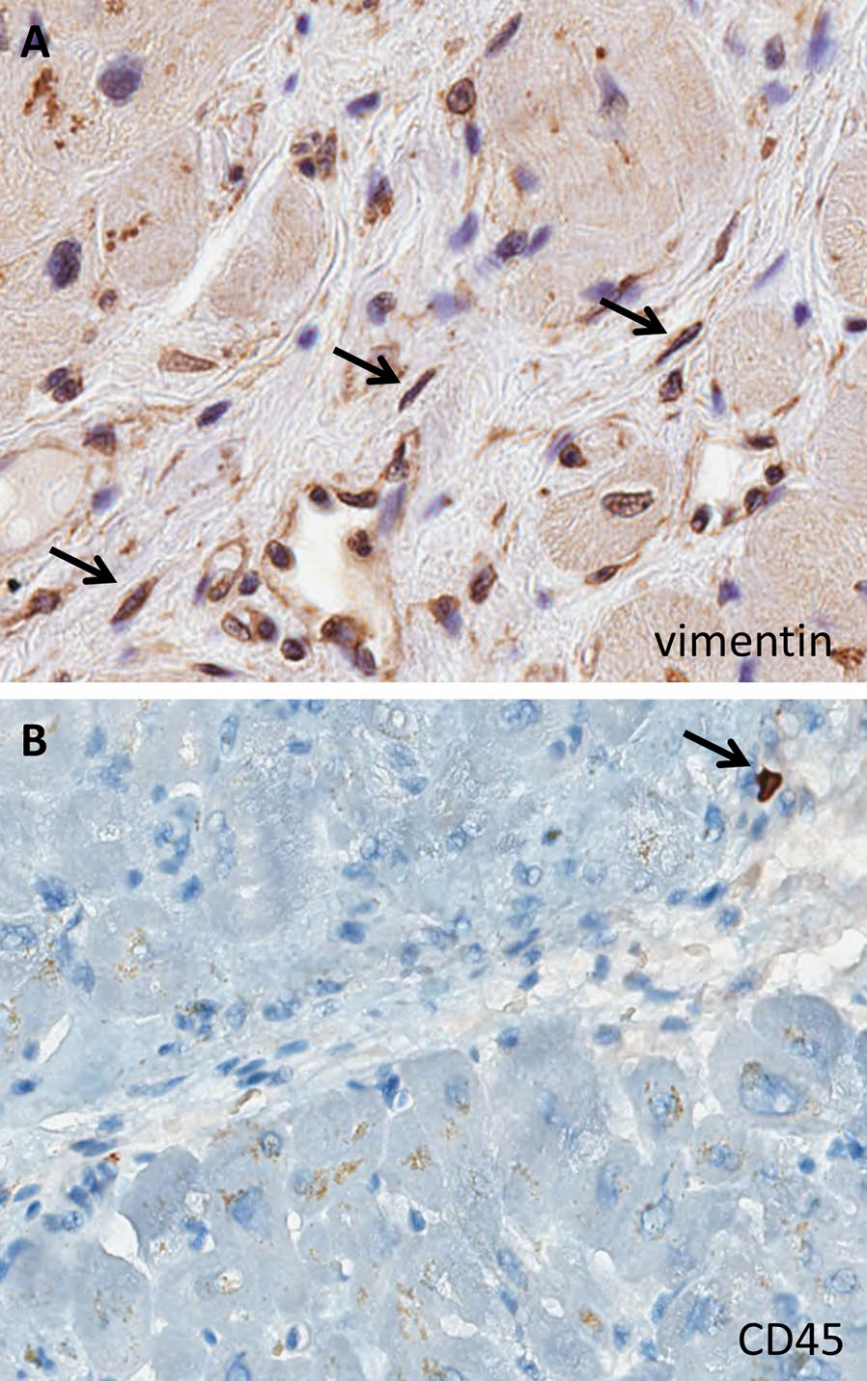
Characterization of Fibrotic Areas (Immunohistochemistry). **a** Myocardial fibrosis is composed by collagen fibers with inter-dispersed vimentin positive fibroblasts (*arrows*). **b** Only rare CD45 positive inflammatory cells are encountered (*arrow*) (original magnification, ×10)

To distinguish fibroblasts from inflammatory cells, immunohistochemistry was performed on myocardial fibrosis for vimentin and CD45. Figure 1 clearly showed that myocardial fibrosis was composed not only by collagen fibres, highlighted by Masson’s Trichrome, but also by fibroblasts (vimentin positive); on the contrary only few inflammatory cells (CD45 positive) were present.

**Fig. 2:**
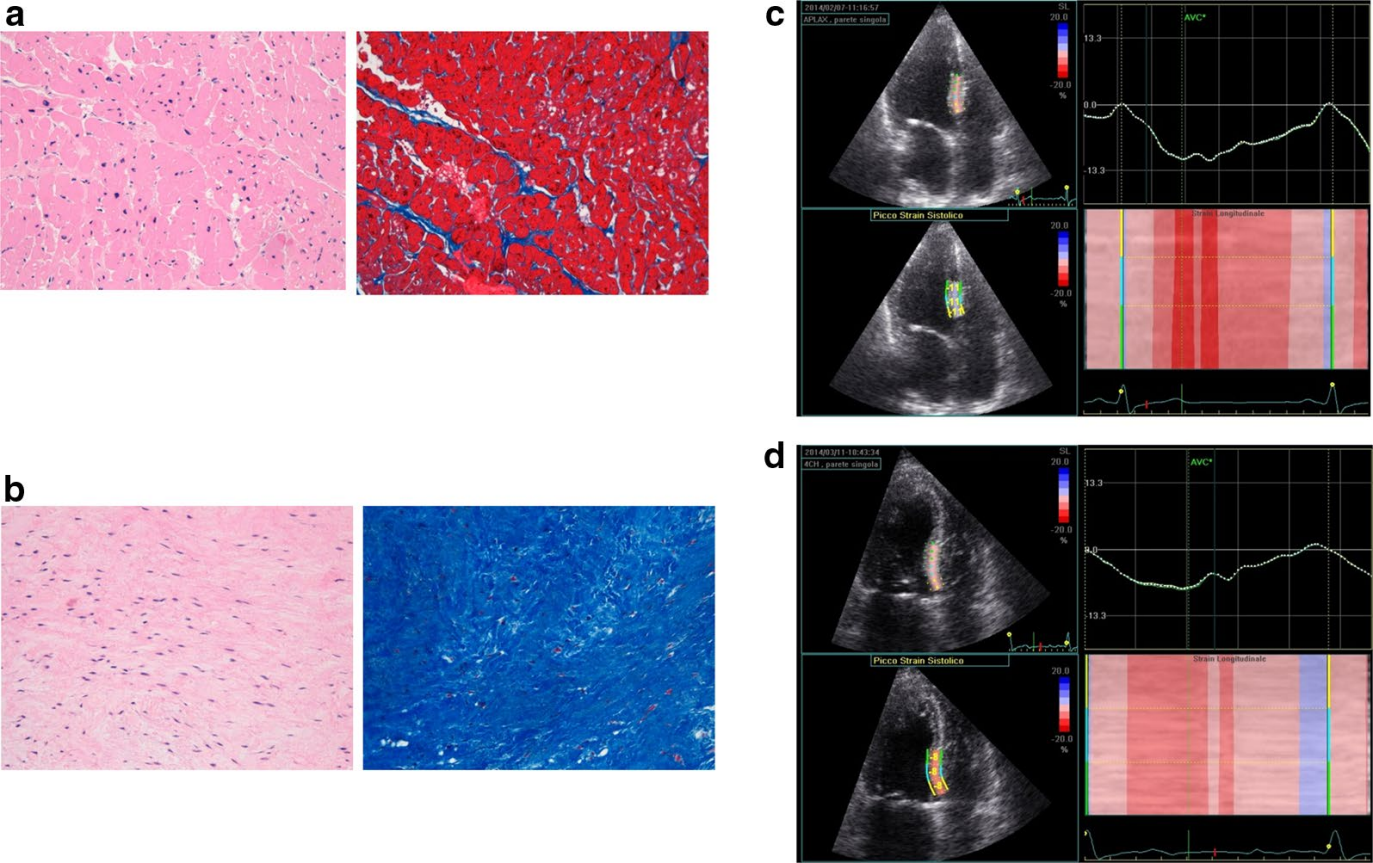
Tissue samples. Samples from intra-operatory biopsies (**a** low and **b** high myocardial fibrosis at basal interventricular septum level), showing myocardial fibrosis (Hematoxylin Eosin/Masson’s Trichrome). Region of interest (ROI) traced to derive longitudinal septal strain values (SSL %; **c** −11 %/**d** −8 %) are shown

MF % was associated (direct relationship) with EDPi (R^2^ = 0.31; p = 0.03) and showed an inverse relationship with SVi (R^2^ = 0.23; p = 0.02). Moreover, MF showed a significant inverse relationship with deformation indices (GLS: R^2^ = 0.30 and p = 0.02; SSL: R^2^ = 0.36 and p = 0.01; SL-Sr: R^2^ = 0.39 and p < 0.001; SL-SrE: R^2^ = 0.35 and p = 0.001) (in Fig. 2 two samples)

We did not find any relevant association between MF and other measures of LV systolic function (i.e. FE, MAPSE or TDI systolic velocities). Moreover, MF showed no association with afterload parameters, including AVAi, Z_VA_ and gradients. No relationships were found between MF and the major clinical and demographical parameters, such as age, duration of the disease, gender, history of diabetes mellitus, dyslipidemia, arterial hypertension and obesity.

### Tissue and plasmatic miRNA-21 analysis

**Fig. 3:**
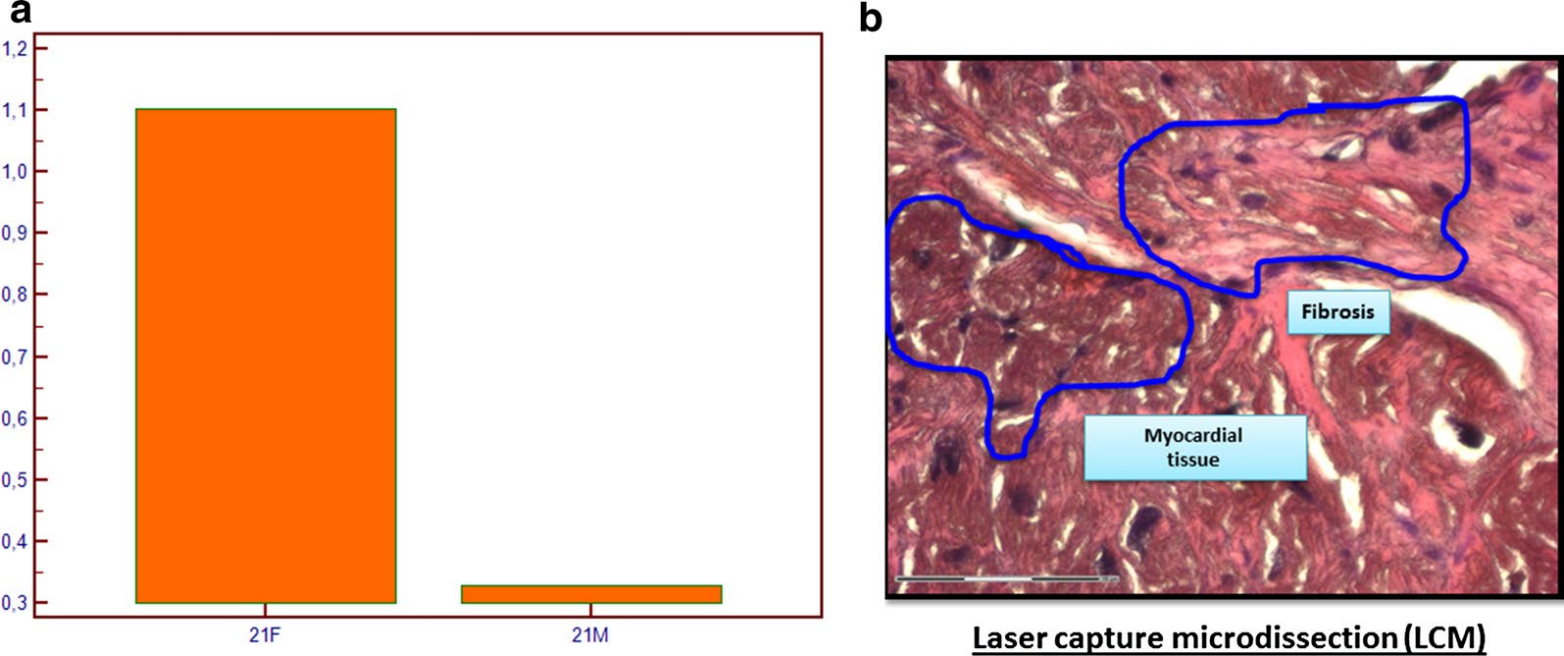
miRNA expression in tissue samples. Differential levels of expression of miRNA-21 in myocardial and interstitial tissue. The levels of expression (**a**) of miRNA-21 in the interstitial compart normalized for the area of fibrosis (*21F*) resulted higher (p < 0.0001) than in myocardial compart, normalized for the myocardial area of the specimen (*21M*). In (**b**) a picture from a specimen

While miRNA-21 was expressed both in myocytes and interstitial tissue, it resulted significantly more expressed in fibrous tissue (p < 0.0001; Fig. 3). Tissue miRNA-21 levels did not show an association with LVMi, body mass index (BMI), BSA or age. Conversely, interstitial miRNA-21 was inversely related to septal and global longitudinal deformation (SSL: R^2^ = 0.32 and p = 0.01; GLS: R^2^ = 0.34 and p = 0.008). Plasmatic miRNA-21 concentrations (n = 30) demonstrated a significant direct relationship with whole MF (R^2^ = 0.31; p = 0.001) and interstitial miRNA-21 compartment (R^2^ = 0.36; p = 0.001).

No relationships were found between plasmatic or tissue miRNA-21 and the major clinical and demographical parameters.

### Integrated speckle tracking and plasmatic miRNA-21 analysis

**Fig. 4:**
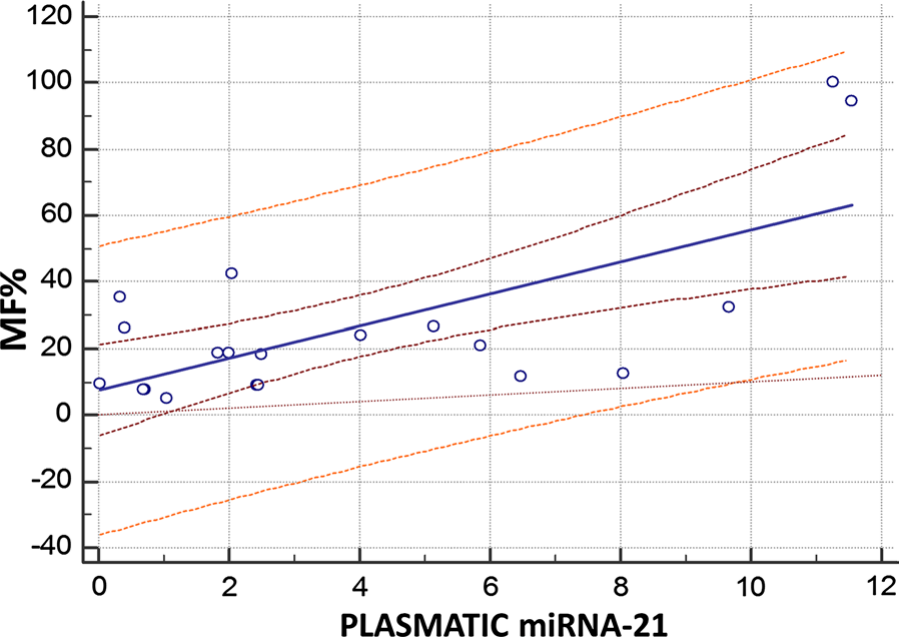
Univariate regression (including 95 % confidence, prediction and line of equality) weighted for Left Ventricular Mass_i_. Myocardial fibrosis in percentage (MF %) and plasmatic levels of micro-RNA-21 (miRNA-21): R^2^ = 0.50; p = 0.0005

A significant and strong positive relationship between MF and plasmatic miRNA-21 was found, also after weighting for cardiac remodeling (assessed as LVMi: R^2^ = 0.50; p = 0.0005) and LV function parameters (SSL R^2^ = 0.35; p = 0.006; Fig. 4).

**Fig. 5:**
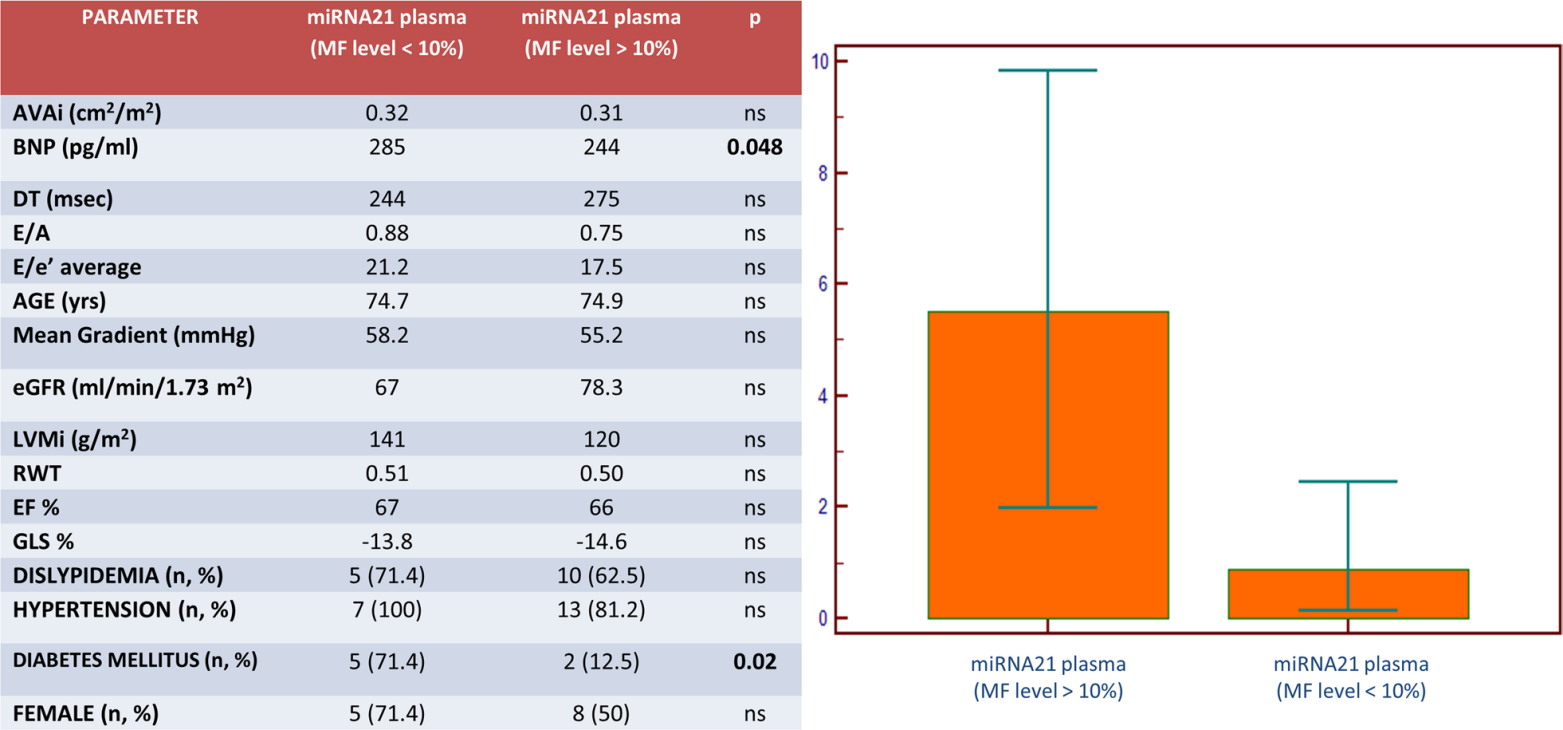
Differential levels of expression of plasmatic miRNA-21 in patients with significant MF (MF % > 10 %). The plasmatic levels of miRNA-21 in patients with high MF (Over Ten = 1) resulted higher respective to the low fibrosis group (Median 5.5043 vs. 0.8854; p = 0.03). The table shows principal differences in clinical profiles. *AVAi* indexed aortic valve area, *BNP* brain natriuretic peptide, *DT* deceleration time, *E/A* ratio of early to late diastolic mitral filling velocity (PW), *EF* ejection fraction, *eGFR* estimated glomerular filtration rate, *GLS* global longitudinal strain, *LVMi* indexed left ventricular mass, *MF* Myocardial Fibrosis, *RWT* relative wall thickness

Neither GLS nor SSL showed a significant diagnostic accuracy in MF evaluation using c-statistic approach. Conversely, patients with higher MF, showed a significant higher mean level of expression of plasmatic miRNA-21. (Figure 5; with table showing differences in clinical profile).

At ROC analysis, a plasmatic miRNA-21 value >2.4552 showed the best accuracy (Sensitivity 64.29 %; Specificity 100 %; AUC 0.81; p = 0.001) for discriminating patients with significant MF (described as equal or more than 10 % of the specimen).

No gender-based differences were found in the study.

## Discussion

The main findings of the present paper are:

- Patients with severe AVS show abnormalities of regional and global left ventricular myocardial strain, reflecting both pressure overload and geometric remodeling. These deformation abnormalities are related with the level of invasively measured MF (gold standard);
- The expression of textural miRNA-21 determined with laser micro-dissection may document its pathophysiological role in AVS. In particular, we focused on interdependence between textural miRNA-21 and fibrogenic stimulus induced by an abnormal left ventricular pressure overload;
- Circulating miRNA-21 (biomarker) levels are high in patients with severe AVS, reflecting the presence of significant myocardial fibrosis (defined as MF % higher or lower than 10 %).

### Deformation imaging and myocardial fibrosis in aortic stenosis

Conditions of LV pressure overload determine a deep remodeling of the extracellular matrix, with the secondary deposition of MF. In the clinical setting, MF is known to be a deleterious consequence of AVS, contributing to systo-diastolic alterations and arrythmogenicity, affecting patients’ prognosis and quality of life after AVR. [27]

Meanwhile, we should not forget that in AVS, the paradigm “Pressure overload—LV remodeling—Myocardial hypertrophy—interstitial and later replacement fibrosis” remains still not so definite: indeed, there is a wide variation, independent from the stage of the disease (especially if we consider only Valve Area). Thus, some patients with severe AVS have normal ventricular structure and no/mild fibrosis (10–30 %) while patients with only moderate AVS may have extensive hypertrophy and large amount of fibrosis [13, 25–27].

In our study population, despite a preserved EF, we found a significant amount of MF at endo-myocardial biopsy, confirming the insensitivity of EF in revealing subtle myocardial textural alterations. On the contrary, as previously reported, myocardial deformation parameters, assessed by 2D-STE, were altered (in a context of preserved EF) and inversely related to global afterload and remodeling parameters (LVMi) [12, 28, 29]. Similarly, we found a significant association between GLS and stroke volume, an important index for AVS re-classification and management [4]. Moreover, myocardial deformation indices showed a significant inverse association with tissue MF, offering the potential appeal of a non-invasive, cost-effective (respective to MRI) tool for the detection of MF and for better AVS risk stratification.

In attempting to estimate MF, many non-invasive imaging modalities showed good correlation with tissue data [30, 31]. To our knowledge, while previous reports have shown a correlation between longitudinal echo parameters (MAPSE; strain TDI) [25, 26] or reflectivity indices (IBS) [32] and MF in AVS patients, the possible relationship between the presence and extent of MF and novel, more sensitive, echocardiographic parameters (i.e. 2D-STE) has never been addressed before.

In our study, we did not find a significant association between MAPSE or TDI (systolic velocities) and textural parameters. These results are in line with recent studies conducted in patients with Hypertrophic Cardiomyopathy that underwent septal miectomy. In fact, deformation parameters showed a strict correlation with myocardial fibrosis, while there was no association between MF and conventional echo parameters, including TDI [33, 34]. In addition, recent T1-mapping MRI techniques, found a significant correlation between the signal and MF and between signal intensity and deformation indices (GLS). [35, 36]

### microRNA and aortic valve stenosis

Recent evidences showed a key role for miRNAs, including miRNA-21, in cardio-vascular pathophysiology. However, only few recent papers evaluated their involvement in human heart, considering plasmatic and (seldom) tissue pools [15]. Several previous findings have underlined the fibrogenic potential of miRNA-21 in hearts with superimposed pressure overload, mediating mRNA for fibrillar proteins. miRNA-21 already showed a pathophysiological role in AVS, with plasmatic levels resulting higher respective to controls and correlating with mean valvular gradient. [37]

To our knowledge, this is the first paper evaluating tissue miRNA expression in biopsies of patients (in vivo) with AVS with LSMD, a more precise method respective to in situ hybridation (FISH).

In line with previous findings, tissue expression of miRNA was higher for interstitial compartment than myocardial tissue [37], with no relationship with myocardial mass. The reason is probably because LVM reflects both myocardial and fibrous tissue compartment, while miRNA-21 is mainly limited to fibrous compartment, potentially (as we may argue from immunohistochemical findings) derived from fibroblasts. Interestingly, miRNA-21 levels of expression in fibrous tissue, in line with their absolute over-expression at that level, showed a significant inverse association with deformation indices. Most important, for plasmatic levels of miRNA-21, which already resulted elevated in previous cohorts of patients with AVS respective to controls [37–39], we found a direct association with MF and interstitial miRNA-21 levels. This finding was stronger if weighted for LVMi (a gross indicator of “whole remodeling”) value. Thus, after validation in larger cohorts, plasmatic levels of miRNA-21 could be used as a reliable biomarker of myocardial fibrosis [40].

Recently, miRNA plasmatic levels confirmed their strong fibrogenic implications in other similar contexts [41].

This may also open, in perspective, to myocardial fibrosis inhibition targeting, as very recently shown by Gupta et al. in animal models of acute allograft cardiac transplantation. [42]

### Putting together the puzzle: dual-step functional and textural analysis

We propose a complementary role of echocardiographic speckle tracking and plasmatic miRNA-21 analysis: the identification of the remodeling process (at macroscopic and tissue level) combined with a refined functional approach. In particular, the evaluation of plasmatic miRNA together with GLS (by summing functional, structural and textural parameters) could help in better stratifying those patients that currently fall in a diagnostic “gray zone” of AVS severity. Then, we can speculate a potential clinical implication in terms of clinical practice/safety (i.e. myocardial biopsy) and cost reduction (e.g. if we consider during the follow up other expensive imaging procedures, such as magnetic resonance imaging). Present results underscore the tight relationship between valve and myocardium (in our opinion the “main actor” of this complex pathophysiological process), suggesting that only a combined evaluation of both variables may allow a complete evaluation of patients with AVS [7].

## Limitations

The present work was designed as a pilot study. The main limitation of the study is its small sample size and, at present, the absence of a follow-up. Analysis of plasmatic miRNA-21 is promising, but must be validated in larger studies, as its prognostic role and remodeling implications. To define a significant amount of MF evaluated with myocardial biopsy, we arbitrarily decided the cut-off of 10 %, according to the literature [13, 24].

So far, above all due to the limited cases collected, we didn’t have the objective to derive cut-off for plasmatic/tissue values of miRNA-21.

Finally, this study was not designed to identify risk-factors associated with MF in patients with AVS. Therefore, it is possible that the small number of patients fails in showing significant differences between subjects with similar age and cardio-vascular risk profiles (diabetes, hypertension etc.). Anyway, consistent with previous and larger observations [25–27], the degree of hypertrophy/MF is not strictly associated with cardio-vascular risk profile. Genetic factors and gender are likely to play an important role in modulating myocardial response. This may explain the large inter-individual variability in remodeling and fibrosis observed in the setting of AVS.

## Conclusions

In patients with severe AVS, myocardial fibrosis was associated with significant alterations of both plasmatic and textural miRNA-21 (biomarker) levels, as well as with impairment of regional and global longitudinal strain (functional marker). This combined bio-humoral and functional evaluation could allow a better definition of the remodeling process that takes place in AVS, possibly further improving risk stratification of patients. Prospective studies in larger populations of patients with AVS, are needed to better analyze the effective prognostic value of this imaging and bio-humoral integrated approach, in order to shift the clinical focus also on myocardium, beside valvular apparatus.

## Abbreviations

2D-STE: 2D-speckle tracking echocardiography
AVAi: indexed aortic valve area
AVS: aortic valve stenosis
BMI: body mass index
BNP: brain natriuretic peptide
BSA: body surface area
cDNA: copied DNA
CI: cardiac index
DT: deceleration time
E/A: ratio of early to late diastolic mitral filling velocity (PW)
EDPi: invasive left ventricular end-diastolic pressure
EF: ejection fraction
fps: frame per second
FISH: fluorescence in situ hybridation
GLS: global longitudinal strain
hs-TnT: high sensitivity assay troponin-T
IBS: integrated back-scatter
ICC: intra/inter-class correlation coefficient
LAVi: indexed left atrial volume
LSMD: laser micro-dissection
LV: left ventricular
LVH: left ventricular hypertrophy
LVMi: indexed left ventricular mass
MAPSE: mitral annular plane systolic excursion
MF: myocardial fibrosis
miRNA: microRNA (ribonucleic acids, RNA)
MRI: magnetic resonance imaging
NYHA: New York Heart association
PCR: polymerase chain reaction
ROI: region of interest
RWT: relative wall thickness
s’L: systolic velocity (TDI) lateral
s’S: systolic velocity (TDI) septal
sAVR: surgical aortic valve replacement
SNORD: small nucleolar RNA
SSL: septal longitudinal strain
SL-Sr: septal longitudinal strain rate
SL-SrE: septal early-diastolic longitudinal strain rate
SVi: indexed stroke volume
TDI: tissue Doppler imaging
Z_VA_: valvulo-arterial impedance
